# Significance of Dietary Antioxidants for Health

**DOI:** 10.3390/ijms13010173

**Published:** 2011-12-23

**Authors:** Michael H. Gordon

**Affiliations:** Institute of Cardiovascular and Metabolic Research and Hugh Sinclair Unit of Human Nutrition, Department of Food and Nutritional Sciences, University of Reading, Whiteknights P.O. Box 226, Reading RG6 6AP, UK; E-Mail: m.h.gordon@reading.ac.uk; Tel.: +44-118-3786723; Fax: +44-118-3787708

**Keywords:** antioxidants, bioavailability, health

## Abstract

Since evidence became available that free radicals were involved in mechanisms for the development of major diseases, including cardiovascular disease and cancer, there has been considerable research into the properties of natural dietary antioxidants. However, it has become clear that dietary antioxidants can only have beneficial effects *in vivo* by radical scavenging or effects on redox potential if they are present in tissues or bodily fluids at sufficient concentrations. For many dietary components, absorption is limited or metabolism into derivatives reduces the antioxidant capacity. For many dietary phytochemicals, direct antioxidant effects may be less important for health than other effects including effects on cell signalling or gene expression *in vivo*.

## 1. Introduction

It has been recognised that reactive oxygen species (ROS) generated by mitochondria, phagocytes, peroxisomes, and cytochrome P450 enzymes may cause damage to cellular DNA, proteins and lipids. The antioxidant hypothesis was a development from the lipid hypothesis, which related risk of developing cardiovascular disease (CVD) to high saturated fat intake and elevated plasma cholesterol levels [1,2]. Intervention trials failed to correlate reduction of plasma cholesterol levels by diet with reduction in cardiovascular disease, so the antioxidant hypothesis focussed attention on the potential for dietary antioxidants to reduce the impact of ROS. It provided an explanation for the reduction in risk of cardiovascular disease arising from increased consumption of fruit and vegetables which has been confirmed by meta analysis [3]. The antioxidant hypothesis proposed that low-density lipoprotein (LDL) cholesterol penetrates the endothelial wall into the sub-endothelial space, where it is susceptible to oxidation by free radicals, smooth muscle cells or macrophages. Oxidised LDL cholesterol is not recognised by the LDL receptors, so it is not cleared from the circulation but it is preferentially taken up by macrophages which become engorged and develop into foam cells. Toxic products are deposited into cells, and fatty streaks form from the foam cells, which develop into atherosclerotic plaques, and consequently play a role in the aetiology of CVD. Vitamin E is the main antioxidant in LDL, but carotenoids may also contribute to the stability of the LDL particle. Other antioxidants may play a role by reducing oxidative stress in plasma, and improving retention of antioxidants in LDL or reducing oxidative damage of Apo B-100, which is the protein in the LDL particle.

## 2. Plasma Antioxidant Capacity

There have been many studies of the antioxidant activity of food components. Dietary components with strong antioxidant activity under selected conditions include vitamin C, vitamin E, carotenoids, and flavonoids. The most common antioxidant mechanisms *in vitro* involve hydrogen-atom transfer, electron donation or metal chelation [4], although carotenoids are also effective singlet oxygen quenchers, which can be important in tissues, e.g., skin, where activation of oxygen may occur [5]. Increases in plasma antioxidant capacity or related measures of antioxidant effects of dietary phytochemicals have often been observed [6–8], but effects of foods containing dietary antioxidants on health outcomes cannot be demonstrated to be due to their antioxidant properties [9,10]. Endogenous compounds (glutathione, ubiquinol, uric acid, bilirubin) and enzymes (superoxide dismutase, catalase, glutathione peroxidase) also make major contributions to the detoxification of ROS. Uric acid, which is a product of purine metabolism, contributes 60–70% of plasma antioxidant capacity [11]. It has been shown to act as an intracellular free radical scavenger and it is active in reducing oxidative stress by reacting with ROS including nitric oxide, peroxyl radicals and hydroxyl radicals. An increase in plasma antioxidant capacity was demonstrated in volunteers who consumed apple juice but this was shown to be due to the increase in serum uric acid concentrations and was not due to the presence of antioxidant polyphenols in the juice [12]. However, elevated serum uric acid concentrations are often observed in subjects with chronic heart failure and metabolic syndrome, and serum uric acid has been proposed as an independent predictor for all major forms of cardiovascular death [13].

Uric acid occurs in plasma at concentrations of 200–500 μmol/L, which is much higher than the concentration of dietary antioxidants. Vitamin C is the dietary antioxidant that occurs at highest concentrations in plasma. However, plasma becomes saturated with vitamin C at about 70 μmol/L, which can be achieved by dietary intake of about 200 mg/day. Vitamin C is well absorbed by individuals who have a low plasma concentration, but once plasma becomes saturated, excess vitamin C is excreted. Epidemiological studies have provided evidence that plasma vitamin C concentration is inversely related to risk of CVD and/or all cause mortality, but clinical trials have not provided clear evidence to support the beneficial effects of vitamin C. Although supplementation with vitamin C may not benefit the general population, a large subpopulation has low plasma concentrations of vitamin C [14] and supplementation with vitamin C may be beneficial for individuals with low plasma vitamin C concentrations [15].

### 2.1. Carotenoids

Carotenoids are partially absorbed from food, but their bioavailability varies widely, being dependent on the food matrix, and the carotenoid structure [16]. Absorption of carotenoids involves release from plant cells, and the formation of micelles which requires dietary fat and bile acids [17]. The carotenes are absorbed by passive diffusion through the intestinal brush border membrane into enterocytes, but for xanthophylls, e.g., lutein, absorption is a facilitated process that requires a class b-type 1 scavenger receptor (SR-B1) [18]. Carotenoids are incorporated into chylomicrons and released into the lymphatic system. They are then incorporated into lipoproteins in the liver and released into the blood stream. Absorption of carotenoids is a relatively slow process with peak plasma concentrations reached at up to 24 h after consumption of the food [19].

The effects of dietary supplementation with β-carotene were investigated in two intervention trials namely the CARET (β-carotene and retinol efficacy trial) and the ATBC (α-tocopherol and β-carotene for cancer prevention) study [20]. Both the CARET and the ATBC study were set up to examine the effects of vitamin supplementation on individuals at high risk of developing lung cancer due to smoking or exposure to asbestos. However, the trials provided evidence of increased morbidity and mortality in the vitamin-supplemented group [21].

Lutein and zeaxanthin are the only carotenoids that occur in the eye, where they are particularly concentrated in the centre of the retina, the macula lutea. They have been identified as being important for resistance to macular degeneration of the retina [22]. Eyes are constantly exposed to ROS, which may be formed due to the influence of UV radiation, which can pass through the cornea and enter the lens. The effect of the carotenoids in absorbing light in the blue and UV region without generating ROS, and in acting as antioxidants by combining with ROS to reduce their activity, makes an important contribution to maintaining the integrity of the retina.

### 2.2. Flavonoids

There has been much interest in dietary flavonoids in recent years. Flavonoids have a common structure consisting of 2 aromatic rings (A and B) that are linked together by 3 carbon atoms, which form an oxygenated heterocyclic ring (ring C) for most flavonoids, although the linking carbons may be in an open chain form for anthocyanins under some conditions. Flavonoids are divided into 6 subclasses: flavonols, flavones, flavanones, flavanols, anthocyanins, and isoflavones. Individual differences within each group arise from the variation in the number and arrangement of the hydroxyl groups and their alkylation and/or glycosylation. Flavonoids are highly active as antioxidants *in vitro* by hydrogen atom transfer, electron donation or metal chelation [4]. Many flavonoids are poorly absorbed from food [23,24]. In the small intestine, flavonoid glucosides may be partly converted to the aglycone by hydrolysis by lactase phlorodzin hydrolase in the brush-border and they may then enter cells by passive diffusion [23]. Alternatively, flavonoid glucosides may be transported into cells, and then hydrolysed by cytosolic β-glucosidase [23]. However, a considerable fraction of ingested flavonoids passes into the large intestine, where colonic microbiota cleave conjugates, and form phenolic acids and hydroxycinnamates. Isoflavones including daidzein-7-*O-*glucosides from soya are reported to reach plasma concentrations up to 3 μM, but other classes reach plasma concentrations < 1 μM after supplementation (Table 1). The nature of the sugar can affect the uptake of dietary flavonoids. Thus quercetin-4′-*O*-glucoside and quercetin-3,4′-*O*-diglucoside from onions reached maximum plasma concentrations in <1 h and 4.7% of intake was recovered in the urine [25]. In contrast when quercetin-3-*O*-rutinoside was consumed in tomato juice, uptake was much slower with time to maximum plasma concentrations *ca.* 5 h, and only 0.02–2.8% of intake was recovered in the urine [26]. These studies confirmed that the absorption of rutinosides in the small intestine was minimal with slower absorption being consistent with absorption from the large intestine. Tea is one of the main sources of flavonoids in the diet. After consumption of green tea, peak plasma concentrations of epigallocatechin gallate, epigallocatechin and epicatechin were reported as 0.04–1 μM, 0.3–5 μM and 0.1–2.5 μM, respectively, in humans [27], but the theaflavins and thearubigins in black tea were less well absorbed with peak plasma concentrations of 2 nM reported for the theaflavins.

The physiological effects of phenolic acids are poorly understood, but effects on gene expression of antioxidant enzymes have been demonstrated. Thus protocatechuic acid, one of the main metabolites of anthocyanins, has been shown to induce the expression of antioxidant, detoxifying enzymes through c-Jun *N*-terminal kinase (JNK)-mediated nuclear factor (erythroid-derived 2)-like 2 (Nrf2) activation [32]. Flavonoids have also been shown to exert effects on cell signalling and gene expression. An extract from cocoa rich in flavonoids has been shown to suppress tumor necrosis factor (TNF-α)-induced vascular endothelial growth factor expression by inhibiting phosphoinositide 3-kinase (PI3K) and mitogen-activated protein kinase kinase-1 (MEK1) activities [33].

In addition, the antioxidant activity of many flavonoids is reduced by metabolism. Flavonoids are derivatised extensively by glucuronidation, methylation, and sulfation in the intestinal mucosa and the liver [34,35]. The antioxidant activity of derivatives is commonly less than that of the parent flavonoid, as shown for methylation or sulfation of quercetin [36].

## 3. Conclusion

Thus, it is clear that many phytochemicals occur at low concentrations in plasma and tissues. They have many physiological effects, and supplementation with dietary antioxidants at doses that give rise to elevated concentrations in plasma and tissues may cause adverse effects. For the flavonoids, antioxidant activity is reduced by metabolism. Effects of dietary antioxidants on cell signalling and gene expression, where effects can be demonstrated at low concentrations, may be more important for health benefits than direct antioxidant activity.

## Figures and Tables

**Table 1:** Bioavailability of selected flavonoids.

Flavonoid	Occurrence	Time to maximum plasma concentration	maximum plasma concentration	Recovery in urine	Reference
Flavan-3-ols e.g. epicatechin, epigallocatechin gallate	Green tea	1.6–2.3 h	50–125 nmol/L	8.1%	[28]
Flavanones— hesperetin-rutinoside, naringeninin-rutinoside	Orange juice	4.4 h	900 nmol/L	17.3%	[29]
Flavonol rutinosides	Tomato juice	5h	<12 nmol/L	0.02–2.8%	[26]
Flavonol glucosides	onions	<1 h	<665 nmol/L	4.7%	[25]
Isoflavones— daidzein-7-*O*-glucosides	soya	8–9 h	<3 μmol/L	20–50%	[24,30]
Anthocyanins Cyanidin-3-galactoside	Chokeberry juice	1.3 h	32 nmol/L	<0.25%	[31]

## References

[1] Duff G.L., McMillian G.C. (1951). Pathology of atherosclerosis. Am. J. Med.

[2] Keys A. (1953). Atherosclerosis: A problem in newer public health. J. Mt. Sinai Hosp. N Y.

[3] Law M.R., Morris J.K. (1998). By how much does fruit and vegetable consumption reduce the risk of ischaemic heart disease?. Eur. J. Clin. Nutr.

[4] Leopoldini M., Russo N., Toscano M. (2011). The molecular basis of working mechanism of natural polyphenolic antioxidants. Food Chem.

[5] Omoni A.O., Aluko R.E. (2005). The anti-carcinogenic and anti-atherogenic effects of lycopene: A review. Trends Food Sci. Technol.

[6] Roberts W.G., Gordon M.H., Walker A.F. (2003). Effects of enhanced consumption of fruit and vegetables on plasma antioxidant status and oxidative resistance of LDL in smokers supplemented with fish oil. Eur. J. Clin. Nutr.

[7] Seidel C., Boehm V., Vogelsang H., Wagner A., Persin C., Glei M., Pool-Zobel B.L., Jahreis G. (2007). Influence of prebiotics and antioxidants in bread on the immune system, antioxidative status and antioxidative capacity in male smokers and non-smokers. Br. J. Nutr.

[8] Young J.F., Dragsted L.O., Haraldsdottir J., Daneshvar B., Kall M.A., Loft S., Nilsson L., Nielsen S.E., Mayer B., Skibsted L.H. (2002). Green tea extract only affects markers of oxidative status postprandially: Lasting antioxidant effect of flavonoid-free diet. Br. J. Nutr.

[9] Gomez-Juaristi M., Gonzalez-Torres L., Bravo L., Vaquero M.P., Bastida S., Sanchez-Muniz F.J. (2011). Beneficial effects of chocolate on cardiovascular health. Nutr. Hosp.

[10] Benzie I.F.F., Wachtel-Galor S. (2010). Vegetarian diets and public health: Biomarker and redox connections. Antioxid. Redox Signal.

[11] Duplancic D., Kukoc-Modun L., Modun D., Radic N. (2011). simple and rapid method for the determination of uric acid-independent antioxidant capacity. Molecules.

[12] Godycki-Cwirko M., Krol M., Krol B., Zwolinska A., Kolodziejczyk K., Kasielski M., Padula G., Grebocki J., Kazimierska P., Miatkowski M. (2010). Uric acid but not apple polyphenols is responsible for the rise of plasma antioxidant activity after apple juice consumption in healthy subjects. J. Am. Coll. Nutr.

[13] Strasak A.M., Kelleher C.C., Brant L.J., Rapp K., Ruttmann E., Concin H., Diem G., Pfeiffer K.P., Ulmer H. (2008). Serum uric acid is an independent predictor for all major forms of cardiovascular death in 28,613 elderly women: A prospective 21-year follow-up study. Int. J. Cardiol.

[14] Smith J.L., Hodges R.E. (1987). Serum levels of vitamin C in relation to dietary and supplemental intake of vitamin C in smokers and non-smokers. Ann. N.Y. Acad. Sci.

[15] Lykkesfeldt J., Poulsen H.E. (2010). Is vitamin C supplementation beneficial? Lessons learned from randomised controlled trials. Br. J. Nutr.

[16] Yeum K.J., Russell R.M. (2002). Carotenoid bioavailability and bioconversion. Ann. Rev. Nutr.

[17] Tyssandier V., Lyan B., Borel P. (2001). Main factors governing the transfer of carotenoids from emulsion lipid droplets to micelles. Biochim. Biophys. Acta.

[18] Yonekura L., Nagao A. (2007). Intestinal absorption of dietary carotenoids. Mol. Nutr. Food Res.

[19] Novotny J.A., Kurilich A.C., Britz S.J., Clevidence B.A. (2005). Plasma appearance of labeled beta-carotene, lutein, and retinol in humans after consumption of isotopically labeled kale. J. Lipid Res.

[20] Rowe P.M. (1996). CARET and ATBC refine conclusions about beta-carotene. Lancet.

[21] Thurnham D.I. (2007). Macular zeaxanthins and lutein—a review of dietary sources and bioavailability and some relationships with macular pigment optical density and age-related macular disease. Nutr. Res. Rev.

[22] Pryor W.A., Stahl W., Rock C.L. (2000). Beta carotene: From biochemistry to clinical trials. Nutr. Rev.

[23] Del Rio D., Borges G., Crozier A. (2010). Berry flavonoids and phenolics: Bioavailability and evidence of protective effects. Br. J. Nutr.

[24] Manach C., Williamson G., Morand C., Scalbert A., Remesy C. (2005). Bioavailability and bioefficacy of polyphenols in humans. I. Review of 97 bioavailability studies. Am. J. Clin. Nutr.

[25] Mullen W., Edwards C.A., Crozier A. (2006). Absorption, excretion and metabolite profiling of methyl-, glucuronyl-, glucosyl- and sulpho-conjugates of quercetin in human plasma and urine after ingestion of onions. Br. J. Nutr.

[26] Jaganath I.B., Mullen W., Edwards C.A., Crozier A. (2006). The relative contribution of the small and large intestine to the absorption and metabolism of rutin in man. Free Radic. Res.

[27] Lambert J.D., Hong J., Lu H., Meng X., Lee M.-J., Yang C.S., Jain N.K., Siddiqi M., Weisburger J. (2006). Bioavailabilities of Tea Polyphenols in Humans and Rodents. Protective Effects of Tea on Human Health.

[28] Stalmach A., Mullen W., Pecorari M., Serafini M., Crozier A. (2009). Bioavailability of *C*-linked dihydrochalcone and flavanone glucosides in humans following ingestion of unfermented and fermented rooibos teas. J. Agric. Food Chem.

[29] Mullen W., Archeveque M.-A., Edwards C.A., Matsumoto H., Crozier A. (2008). Bioavailability and metabolism of orange juice flavanones in humans: Impact of a full-fat yogurt. J. Agric. Food Chem.

[30] Rufer C.E., Bub A., Moseneder J., Winterhalter P., Stuertz M., Kulling S.E. (2008). Pharmacokinetics of the soybean isoflavone daidzein in its aglycone and glucoside form: A randomized, double-blind, crossover study. Am. J. Clin. Nutr.

[31] Wiczkowski W., Romaszko E., Piskula M.K. (2010). Bioavailability of cyanidin glycosides from natural chokeberry (*Aronia melanocarpa*) juice with dietary-relevant dose of anthocyanins in humans. J. Agric. Food Chem.

[32] Vari R., D’Archivio M., Filesi C., Carotenuto S., Scazzocchio B., Santangelo C., Giovannini C., Masella R. (2011). Protocatechuic acid induces antioxidant/detoxifying enzyme expression through JNK-mediated Nrf2 activation in murine macrophages. J. Nutr. Biochem.

[33] Kim J.-E., Son J.E., Jung S.K., Kang N.J., Lee C.Y., Lee K.W., Lee H.J. (2010). Cocoa polyphenols suppress TNF-α-induced vascular endothelial growth factor expression by inhibiting phosphoinositide 3-kinase (PI3K) and mitogen-activated protein kinase kinase-1 (MEK1) activities in mouse epidermal cells. Br. J. Nutr.

[34] Manach C., Scalbert A., Morand C., Remesy C., Jimenez L. (2004). Polyphenols: Food sources and bioavailability. Am. J. Clin. Nutr.

[35] Yang C.S., Sang S., Lambert J.D., Lee M.J. (2008). Bioavailability issues in studying the health effects of plant polyphenolic compounds. Mol. Nutr. Food Res.

[36] Lotito S.B., Zhang W.-J., Yang C.S., Crozier A., Frei B. (2011). Metabolic conversion of dietary flavonoids alters their anti-inflammatory and antioxidant properties. Free Radic. Biol. Med.

